# Estrogen Sulfotransferase (SULT1E1): Its Molecular Regulation, Polymorphisms, and Clinical Perspectives

**DOI:** 10.3390/jpm11030194

**Published:** 2021-03-11

**Authors:** MyeongJin Yi, Masahiko Negishi, Su-Jun Lee

**Affiliations:** 1 Pharmacogenetics Section, Reproductive and Developmental Biology Laboratory, National Institute of Environmental Health Sciences, National Institutes of Health, Research Triangle Park, NC 27709, USA; myeongjin.yi@nih.gov (M.Y.); negishi@niehs.nih.gov (M.N.); 2 Department of Pharmacology and Pharmacogenomics Research Center, Inje University College of Medicine, Inje University, Bokji-ro 75, Busanjin-gu, Busan 47392, Korea

**Keywords:** estrogen sulfotransferase, SULT1E1, estrogen, estrogen sulfate, thyroid hormones, breast cancer, endometrial cancer, polymorphism

## Abstract

Estrogen sulfotransferase (SULT1E1) is a phase II enzyme that sulfates estrogens to inactivate them and regulate their homeostasis. This enzyme is also involved in the sulfation of thyroid hormones and several marketed medicines. Though the profound action of SULT1E1 in molecular/pathological biology has been extensively studied, its genetic variants and functional studies have been comparatively rarely studied. Genetic variants of this gene are associated with some diseases, especially sex-hormone-related cancers. Comprehending the role and polymorphisms of SULT1E1 is crucial to developing and integrating its clinical relevance; therefore, this study gathered and reviewed various literature studies to outline several aspects of the function, molecular regulation, and polymorphisms of SULT1E1.

## 1. Introduction

The metabolism of endogenous compounds and hormones is important in physiological homeostasis. Sulfation, which occurs in many metabolic pathways, generates sulfoconjugated forms that are typically regarded as inactive metabolites. Sulfation is one of the phase II metabolizing pathways that represent the inactivation of hormones, such as estrogens, and this reaction is usually performed by an enzyme from the cytosolic enzyme group referred to as sulfotransferases (SULTs) [1]. In various mammals, such as mice and rats, SULTs play the essential roles of sulfating estrogens, thyroid hormones, bile acids, and other xenobiotics [2,3,4,5,6].

Thirteen cytosolic SULTs have been identified in humans (Table 1). Sulfotransferases facilitate the SN_2_-like displacement/transfer reaction of a sulfonate (SO_3_^−^) group from the ubiquitous donor 3′-phosphoadenosine 5′-phosphosulfate (PAPS) to hydroxyl or amino residues of acceptor substrates [7,8]. Many active sites of SULTs are conserved; they are the same in all known crystal structures of human and mouse SULTs [9,10,11,12,13].

Sulfotransferase isoforms sulfate not only xenobiotics (e.g., flavonoids and hydroxyl metabolites of anticancer drugs) but also endogenous compounds, such as steroid hormones. They have essential roles in the homeostasis of bile acids, thyroid hormone, androgens, and estrogens, and their expressions are influenced by substrates and pathological conditions as well [14].

Among the SULT isoforms, SULT1E1 has the lowest *K_m_* values for estrone (E_1_), estradiol (E_2_), and catecholestrogen sulfation [15,16,17,18,19]. This enzyme had been referred to as EST (estrogen sulfotransferase) due to its substantial role in estrogen inactivation. It was discovered and cloned in other mammalian species (Table 2), and its amino acid sequence homologies regarding rabbit, horse, pig, mouse, cow, and rat EST compared with human EST are 82.6%, 79.2%, 77.8%, 77.5%, 73.7%, and 71.3%, respectively.

Although the SULT1A subfamily can sulfate estrogens, their affinity for endogenous estrogens is significantly lower than the affinity of SULT1E1 for those substrates. Moreover, SULT1E1 engages in the sulfation of thyroid hormones alongside the SULT1A subfamily. Though the important roles and regulation of SULT1E1 have been identified and stressed, functional studies related to genetic variants are relatively limited.

This review concentrates on the expression, functional characterization, regulation, associations with diseases, and genetic polymorphisms of SULT1E1.

## 2. Expression of SULT1E1

Human SULT1E1 cDNA was first isolated, cloned, and characterized from the liver, and its localization was mapped to human chromosome 4 [20]. SULT1E1 is expressed in the human embryo, and is also highly expressed in a wide range of fetal tissues, such as the liver, lung, kidney, and hormone-dependent tissues—such as the testis or endometrium—but its expression in adults with normal status is much lower than in the fetus and placenta [21,22]. The expression of SULT1E1 varies widely in the human population, although it is not known whether this is under genetic control or not [23]. Thus, it is possible that the variability in SULT1E1 expression results from different chemical influences.

Two agonists of peroxisome-proliferator-activated receptor α (PPARα), WY14643 and IGF-1, show different regulatory effects on the SULT1E1 promoter activity. While WY14643 suppressed SULT1E1 activity, IGF-1 upregulated it, as measured by estrogen levels in endothelial cells and smooth muscle cells [24]. Interestingly, SULT1E1 was attenuated by both transfection with PPARγ small interfering RNA (siRNA) and exposure to GW9662, the PPARγ antagonist [25].

SULT1E1 regulation was observed when hepatocyte nuclear factor 4α (HNF4α) was silenced. The significant suppression of both mRNA and protein levels of SULT1E1 occurred via Farnesoid X receptor (FXR) agonists in HepG2 cells [26]. This finding confirmed that the effect of FXR on E_2_ was SULT1E1-dependent. In patients with obstructive cholestasis, the accumulation of bile acids (activator of FXR) led to reduced mRNA and protein expression of hepatic SULT1E1, increased serum E_2_ levels, and decreased serum estrone sulfate concentration [27]. Phosphorylated RORα takes a regulatory signal to HNF4α, and then activates the *SULT1E1* promoter in human liver cells [28].

Basal expression of SULT1E1 in the liver is relatively low [29], but its expression and role could be impacted in response to ligands/substrates for nuclear receptors, such as the liver X receptor (LXR) [29], the glucocorticoid receptor (GR) [30], the constitutive androstane receptor (CAR) [31], the estrogen receptor α (ERα) [32], the pregnane X receptor (PXR) [33], and the RAR-related orphan receptor α (RORα) [34] (Table 3).

## 3. Sulfation of Estrogens and Thyroid Hormones by SULT1E1

### 3.1. Sulfation of Estrogens

**Figure 1:**
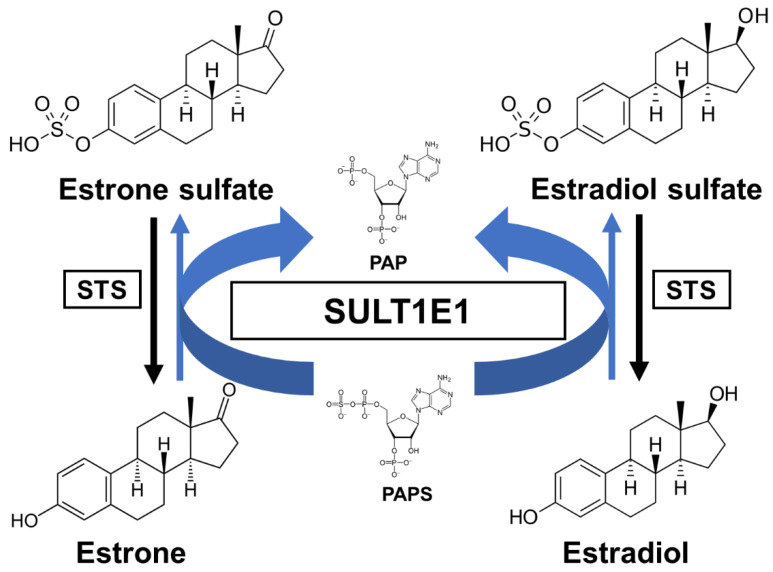
A schematic sulfation pathway of estrogens. STS, steroid sulfatase; PAPS, 3′-phosphoadenosine 5′-phosphosulfate; PAP, 3′-phosphoadenosine 5′-phosphate.

Estrogens play fundamental roles in a variety of physiological systems. It has been widely established that estradiol (E2) exposure is one of the risk factors for breast carcinogenesis. One of the critical pathways for E2 inactivation is sulfation by SULT1E1. Estrone (E1) is synthesized by aromatization of androstenedione and is subsequently sulfated. After E1 is desulfated and subsequently turned into E2 by the 17β-hydroxysteroid dehydrogenases (17β-HSD), E2 can then be sulfated through SULT1E1 [35]. As previously mentioned, SULT1E1 is a cytosolic enzyme that catalyzes estrogen sulfation at the 3-hydroxyl site while using PAPS as a sulfate donor (Figure 1). Moreover, this enzyme has high affinity for its substrate E2, indicating its crucial role in modulating estrogen’s action and homeostasis [36].

SULT1E1 has shown the distinct characteristic of having a high sulfating affinity for not only E_2_, but also other estrogens, such as E_1_ and ethinylestradiol (EE_2_), with nanomolar *K_m_* values (Table 4). Due to its high affinity for sulfate estrogens, SULT1E1 exhibits inhibition of substrate with increasing E_2_ and E_1_ concentrations. SULT1E1 is also used to sulfate other compounds, namely dehydroepiandrosterone (DHEA), pregnenolone, diethylstilbestrol (DES), and equilenin [37,38].

SULT1E1 is also expressed in hormone-dependent tissues, such as endometrium [22,48] and placenta [21]. SULT1E1 is specifically expressed during the secretory phase of the menstrual cycle in human endometrium [49]. Upregulated SULT1E1 activity in the endometrium may result in sulfating E_2_ after ovulation [50]. In addition, SULT1E1 can be induced by progestins in human Ishikawa endometrial adenocarcinoma cells [51].

As an interesting effect, estrogens inhibit expression of the potent growth factor repressor transforming growth factor (TGF)-β1. In addition, it was observed that MCF-7 cells expressing SULT1E1 activity did not show a decrease in ERα levels, an increase in progesterone receptor, or a decrease in transforming growth factor-β expression, suggesting the rapid sulfoconjugation of E_2_ by SULT1E1.

It is possible that SULT1E1 contributes to EE_2_ sulfation during hepatic-mediated first-pass metabolism. SULT1E1 is the high-affinity enzyme responsible for EE_2_ sulfation at nanomolar concentrations, so SULT1E1 plays a predominant role in the sulfation of EE_2_ in the intestine and liver.

### 3.2. Sulfation of Thyroid Hormones

**Figure 2:**
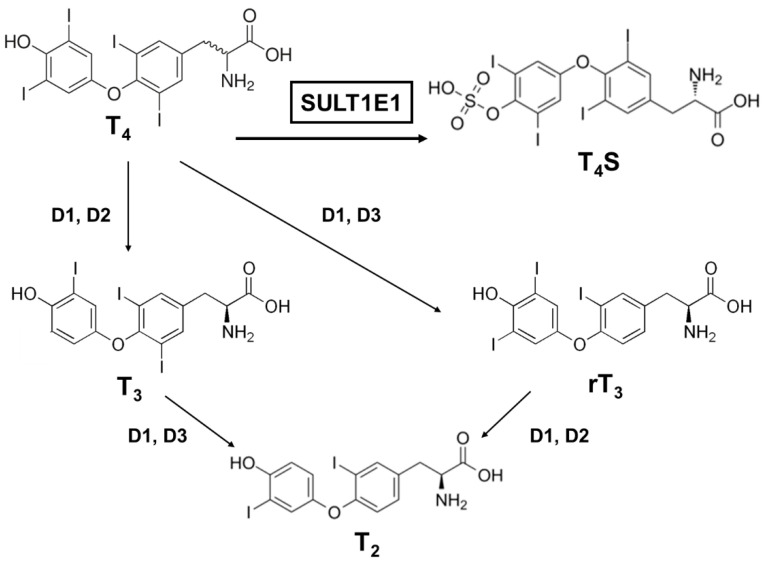
A schematic metabolic pathway of thyroid hormones. T_4_, thyroxine (prohormone); T_4_S, thyroxine sulfate (sulfoconjugated metabolite); T_3_, 3.3′,5-triiodothyronine (receptor active iodothyronine); rT_3_, 3.3′,5′-triiodothyronine (receptor inactive iodothyronine); T_2_, 3.3′-diiodothyronine.

Many factors serve as regulators for the effectiveness and bioavailability of receptor active thyroid hormone (T_3_) [52,53]. The prohormone thyroxine (T_4_) is predominantly secreted to regulate metabolism [54,55]. Deiodination is one of the principal and major pathways to degrade active compounds, and there are three types of deiodinase selenoproteins—iodothyronine deiodinases (D1, D2, and D3) [56]. These deiodinases are promotive of the reductive T_4_ deiodination and its metabolites (Figure 2).

One major modification thyroid hormones receive is sulfation, which deactivates them. Thyroxine sulfate can be detected in human fetal blood and amniotic fluid, indicating that the production of sulfoconjugates is critical in utero [57]. Iodothyronine sulfates (T_4_S, T_3_S, rT_3_S, and T_2_S) are generated by SULT enzymes, which are located in a variety of different tissues, and catalyze the sulfation and substitution of the hydroxyl groups of various compounds using PAPS as the sulfate donor [58]. Interestingly, among the SULTs, most of the SULT1 enzymes catalyze the sulfation of iodothyronines [41,59,60,61]. SULT1E1 is highly effective at catalyzing rT_3_ sulfation and has sulfating activity for all iodothyronines (Table 4). For rT_3_ sulfation especially, SULT1E1 has the highest activity among the SULT1 subfamily, even compared to SULT1A1 [59]. Moreover, SULT1E1 was reported as the most active enzyme that exhibited catalyzing activity for T_4_ sulfation [62].

SULT1E1 can be detected in the human endometrium and in the mouse uterus, so it might be possible that the uterus could protect the fetus from excessive thyroid hormone by inactivating pathways via SULT1E1 or D3. It is notable that the metabolites derived from D3 (rT_3_ and T_2_) are also favorable substrates of SULT1E1, suggesting that T_4_ and T_3_ are metabolized in the uterus by consecutive sulfation. The physiological roles of each iodothyronine SULT still remain too complex to be comprehended in full. Although SULT1E1 has been proven to be a potent iodothyronine SULT along with SULT1A1, it is probable that the other SULT1 enzymes contribute to iodothyronine sulfation in a tissue- or growth-dependent way [63,64,65].

## 4. Sulfation of Other Substrates by SULT1E1

SULT1E1 has the role of sulfotransferase not only for endogenous substrates, such as estrogens or iodothyronines, but also for various other compounds (Table 4).

Flavonoids are a class of naturally occurring polyphenols in most plants, and they play diverse roles. Many of them have antioxidative influences in vitro and in vivo. Apigenin (4′,5.7-trihydroxyflavone) is one of the flavonoids that usually exists in chamomile flowers, and it is a yellow compound that can dye wool [66]. Catechin enantiomers are ubiquitous constituents of herbal medicines. The active isomer (−)-epicatechin is known for its anti-inflammatory effects by the activation of the NF-κB signaling pathway [67]. Resveratrol (3,5,4′-trihydroxy-trans-stilbene) is expressed in several plants in response to damage or attack by pathogens [68]. Chrysin (5.7-dihydroxyflavone) is typically found in honey or propolis [69]. Quercetin (5.7,3′,4′-flavon-3-ol) is distributed in naturally occurring polar auxin transport inhibitor, and it is one of the most common natural dietary flavonoids [70]. Though most polyphenols are sulfated by SULT1A isoforms, many sulfoconjugated forms of polyphenols can be generated by SULT1E1 due to its phenotypic response at the cellular level [42].

Fulvestrant is a novel medicine for endocrine treatment; it is an antagonist of estrogen receptors (ERs) that provides no agonistic activity. This compound is an analog of E_2_ that has a distinguishable structure from nonsteroidal medicines such as tamoxifen and other selective estrogen receptor modulators (SERMs). Fulvestrant performs as a competitive inhibitor and suppresses the binding of E_2_ to the ERs, and SULT1E1 has exhibited clear sulfating activity towards fulvestrant [43].

Synthetic estrogens for oral administration are widely prescribed and given to fertile women. Various SERMs have been developed and administered to inhibit the activation of estrogen’s activity in the breast. It has been revealed that SULT1E1 sulfates 4-hydroxytoremifene (4-OH TOR), an active metabolite of toremifene, alongside SULT1A1 [44]. Among the SULT isoforms, SULT1E1 has a high affinity for the tamoxifen active metabolite 4-hydroxytamoxifen (4-OH TAM) and other active tamoxifen metabolites, including endoxifen and *N*-desmethyltamoxifen (*N*-des TAM), which are substrates of SULT1E1 as well [46]. These metabolites show weak inhibitory effects on SULT1E1, suggesting that they are unlikely to interfere with the sulfation of E_2_ in SULT1E1-expressing tissues.

Troglitazone acts as an agonist of PPARα and has been used as an oral antidiabetic for the treatment of insulin-independent diabetes mellitus. SULT1E1 appropriately sulfates troglitazone and had greater activity than SULT1A1 when 10 uM of troglitazone was treated [45].

After tibolone binds to nuclear receptors, such as ERs, progesterone receptor (PG), and androgen receptor (AR), to activate them, it is dramatically metabolized into two active hydroxylated isomers, 3α-OH and 3ß-OH-tibolone, which can be metabolized into ∆^4^-tibolone. SULT1E1 sulfates tibolone as well as its metabolites, 3α-OH and 3ß-OH-tibolones [47].

## 5. SULT1E1 and Diseases

**Figure 3:**
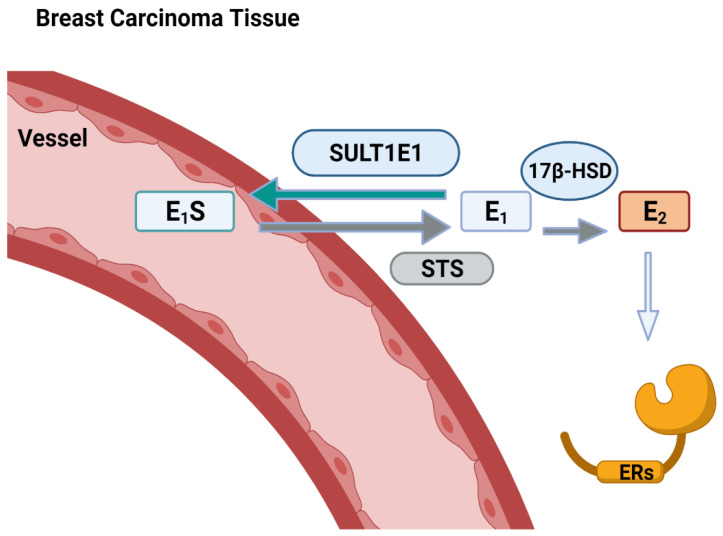
A schematic pathway for estrogen formation by SULT1E1 and STS in breast carcinoma tissue. E_1_S, estrone sulfate; E_1_, estrone; E_2_, estradiol.

Due to SULT1E1 being highly activated in pathophysiological conditions, such as estrogen-related diseases, the quantification of the E_1_S form of estrogen during the menstrual cycle and in menopausal women has been widely used [71,72,73]. It has been reported that a strong association between breast cancer vulnerability and increased E_2_ concentration exists [74]. Moreover, the concentrations of E_1_S and E_2_S are higher in patients with breast fibroadenoma [75] (Figure 3); however, in that same study, the expression of SULT1E1 decreased or was abolished in breast cancer tissues, though it was expressed in normal breast cells. In breast carcinoma cell lines, E_1_ and E_2_ can be sulfoconjugated by SULT1E, which appears to be expressed at low levels in breast cancer cells. The expression of SULT1E1 during the progression of tumorigenesis was characterized using an MCF-7 cell line transfected with SULT1E1, and it was observed that sulfation increased in the SULT1E1-transfected MCF-7 cells compared to the control cells [76]. A similar observation of the physiological implications of SULT1E1 expression was examined by the MCF-7 cell line as well; the response to physiological concentrations of E_2_ was reduced, as determined in an estrogen-responsive reporter gene assay [77]. SULT1E1 has shown very strong affinity for the sulfation of E_2_ and EE_2_, so the ability of SULT1E1 to be involved in estrogen concentrations is important for regulating estrogen receptor target tissues. Estrogen-dependent breast cells with high SULT1E1 levels grow more slowly, suggesting an inhibitory role in carcinogenesis, depending on the role of SULT1E1 in creating physiologically inactive estrogen via sulfoconjugation [51,76,78,79].

Due to the high homology (77.5%) between humans and mice, mouse models have been developed and studied in various approaches. Many pathological mouse models that are related to SULT1E1 have been studied, such as sepsis and diabetes. Sepsis is a lethal condition caused by physiological reactions to infections. There was an in vivo mouse study where hepatic SULT1E1 was upregulated via the activation of the NF-κB pathway’s associated inflammatory pathways [80].

The Akita mouse was derived from C57BL/6J and inherited the mutated insulin 2 gene, so it can be used as a model of diabetes mellitus (DM) type 1. Interestingly, hepatic SULT1E1 mRNA was highly upregulated in Akita, and this pathological situation acts as a stimulus to regulate SULT1E1 expression via phosphorylated-ERα and dephosphorylated-CAR [32]. Likewise, diabetes type 2 mouse models (*db*/*db* and *ob*/*ob*) also exhibited the hepatic overproduction of SULT1E1, representing SULT1E1’s role in maintaining the balance of estrogen sulfation [81,82].

## 6. Functional Variants of SULT1E1 and Current Research Status

A total of 4760 single-nucleotide polymorphisms (SNPs) have been validated by frequency, cluster, and ALFA (allele frequency aggregator) out of the total of 5428 SNPs, including 214 missense variants in human *SULT1E1*, according to NCBI dbSNP. Most SNPs are intronic variants. Diverse studies have been conducted to identify *SULT1E1* polymorphisms and their effects, especially based on association cohort studies (Table 5).

Six SNPs from the introns of *SULT1E1* were associated with treatment failure of abiraterone acetate (AA) therapy in metastatic castration-resistant prostate cancer (mCRPC) patients [83]. Each DNA sample was isolated from patients with mCRPC who were treated with AA approximately three years previously, and the samples were analyzed for the study. In groups 1 (rs3775777, rs4149534, and rs10019305) and 2 (rs3775770, rs4149527, and rs3775768), it was observed that the patients carrying polymorphic alleles had the estimated hazard ratios of 3.58 and 3.12, respectively [83].

There was an association study using Korean females that included breast cancer patients and healthy subjects [84]. The patients carrying rs3775775 (TC or CC) had a hazard ratio of 3.2 (1.39–7.48) compared to that of TT carriers [84]. Regulating estrogen levels, which is especially related to SULT1E1’s sulfation capacity, could facilitate the development of breast cancer or its avoidance in Korean females [84].

The most popular and broadly studied polymorphism of *SULT1E1* is rs3736599, which has a nucleotide alteration at c.–64G>A of the 5′UTR region. Though other variants were also involved, this variant influenced the DHEA sulfation, endometrial carcinogenesis risk, and bone mineral density in females [85,86,87]. There was a cohort study that enrolled equal numbers of African American (AA) and European-American (EA) women, and approximately 11 years after the study’s inception, complete data were collected from 301 women. In the EA women, *SULT1E1* rs3736599 carriers had lower DHEA sulfate levels [85].

In a study in which 150 endometrial cancer patients in total and 165 age-matched healthy control individuals were enrolled [86], surprisingly, the odds ratios of AA and AA+GA were 3.50 and 1.76, respectively, reflecting the higher endometrial cancer risks [86].

In another study, 397 healthy Korean female subjects with menopause and without any cancer or thyroid-related disease history were genotyped to identify the differences in bone mineral density of the distal radius and calcaneus [87]. A variant of *SULT1E1*, rs3736599, was associated with bone mineral density of the distal radius and the calcaneus. Moreover, a combined effect between this polymorphism and altered estrogen consumption might exist in the calcaneus [87].

Three of the *SULT1E1* SNPs—Asp22Tyr (rs11569705), Ala32Val (rs34547148), and Pro253His (rs11569712)—were discovered, and these variants were in the encoded amino acids [88]. These alleles were transfected and expressed in COS-1 cells to discover their functional impacts on stability and activity. Among them, rs11569705 indicated the most significant decrease in enzyme activity and protein level, and rs34547148 also displayed a 50% decrease in both the enzyme and the protein [88].

**Table 5:** Reported human SULT1E1 functional variants.

Type	Position ^1^	SNP ID ^2^	Effect	Reference
Intron	c.772+369T>C	rs3775777	Treatment failure on abiraterone acetate with mCRPC	[83]
	c.369+1930A>C	rs4149534		
	c.369+402T>C	rs10019305		
	c.-9-899G>A	rs3775770		
	c.-10+771C>A	rs4149527		
	c.-10+655G>A	rs3775768		
	c.-9-469G>A	rs3822172	Lower survival rate in colorectal cancer	[89]
	c.772+856G>T,C,A	rs1238574		
	c.369+1653T>C	rs3775775	Decreased survival rate from breast cancer	[84]
5′UTR	c.-64G>A	rs3736599	Lower DHEA sulfate levels in the menopausal transition of European-American population	[85]
			May strongly contribute to risk for endometrial carcinogenesis in Caucasians	[86]
			Higher bone mineral density of distal radius and calcaneus in Korean women	[87]
Missense	95C>T (Ala32Val)	rs34547148	Increased *K_m_* value for the sulfation of E_2_	[88]
	64G>A (Asp22Tyr)	rs11569705		

^1^ All reference sequences are described according to GRCh38.p12 chromosome 4, and the accession number is NM_005420.3. ^2^ Each single-nucleotide polymorphism (SNP) ID is described according to the NCBI dbSNP. UTR, untranslated region; mCRPC, metastatic castration-resistant prostate cancer; DHEA, dehydroepiandrosterone.

## 7. Future Directions for Clinical Integrations

SULT1E1 is responsible for the metabolism of active estrogens and plays crucial roles in their homeostasis. Therefore, this enzyme makes a variety of contributions to human health, including in regard to cancers and drug responses. However, the lack of genetic research on SULT1E1 needs to be enhanced by precisely designed studies in many respects. Several cohort-study-based analyses have been conducted regarding *SULT1E1* genetic variants, but relatively few compared to the number of such studies for the *SULT1A* subfamily.

Due to human SULT1E1’s high nucleotide homology with several animal SULT1E1s, and their similar substrate-binding structures, animal models and in vivo studies have provided useful clues for the genetic regulation and kinetics of humans. Thus, using transgenic animal models would aid in determining gene–gene or gene–xenobiotic interactions in the study of SULT1E1 activity.

Since the substrate-binding sites and neighboring amino acids are regarded as being involved prominently in enzyme activity and structure, we suggest candidate SNPs corresponding to adjacent substrate-binding sites be investigated in genetic association studies (Table 6).

Several cohort studies have developed SULT1E1 association models, such as Predictors of Breast Cancer Recurrence (ProBeCaRE) [90] and U-statistics-based tests for identifying the pathway-based candidate genes of breast cancer and hormone metabolism pathways [91]. In addition, an intronic polymorphism (rs3775779) was discovered as a marker for analyzing the ethnic difference in the fine-scale population structure of Malays in Peninsular Malaysia and Singapore [92]. These studies suggest diverse scientific approaches to figure out the role of SULT1E1.

Many studies of SULT1E1 have highlighted aspects of its impacts on biological systems. Therefore, we encourage such studies to elucidate the related pathophysiological perspectives of human SULT1E1.

**Table 6:** Amino acids near to substrate-binding sites of SULT1E1.

Impacted Amino Acids	Substrate ^1^	Alteration	SNP ID ^2^
Arg256	PAPS	Not reported	-
Phe254	E_2_, 4-OH TCB	Phe254Cys	rs746067466
Met247	4-OH TCB	Met247Ile	rs1188553969
Ile246	4-OH TCB, TBBPA	Ile246Leu	rs1413235220
Tyr239	E_2_, 4-OH TCB	Not reported	-
Phe228	PAPS	Not reported	-
Thr226	PAPS	Thr226Ser	rs756363002
Asn168	4-OH TCB, TBBPA	Asn168Ser	rs1265277815
Val145	4-OH TCB	Val145Leu	rs200443686
Phe141	E_2_, 4-OH TCB, TBBPA, 3-OH BDE47	Phe141Leu	rs1220949195
Phe138	TBBPA	Not reported	-
Ser137	PAPS, E_2_	Ser137Pro	rs1208507410
Arg129	PAPS	Arg129Gln	rs774700339
His107	PAPS, E_2_, 4-OH TCB, TBBPA	His107Arg	rs1316115370
Lys105	PAPS, E_2_, 4-OH TCB, TBBPA, 3-OH BDE47	Not reported	-
Cys83	3-OH BDE47	Cys83Phe	rs1431397129
Phe80	E_2_, 4-OH TCB, TBBPA, 3-OH BDE47	Not reported	-
Trp52	PAPS	Not reported	-
Thr51	PAPS	Thr51Ile	rs1170826222
		Thr51Ala	rs761632873
Thr50	PAPS	Not reported	-
Gly49	PAP	Gly49Val	rs1460190031
		Gly49Ser	rs1210226778
Ser48	PAP	Ser48Cys	rs1336407598
		Ser48Pro	rs1052854963
Lys47	PAPS, E_2_	Lys47Glu	rs1361781887
Pro46	4-OH TCB, TBBPA	Pro46Leu	rs771011878
Phe23	4-OH TCB	Phe23Cys	rs1400776691
Asp22	4-OH TCB	Asp22Asn	rs11569705
		Asp22Tyr	
Tyr20	PAP-E_2_, 4-OH TCB, TBBPA	Tyr20Cys	rs778407495

^1^ The crystal structures and neighboring amino acids of SULT1E1 substrate-binding sites were described according to the RCSB protein data bank (PDBid: 1G3M, 1HY3, 4JVM, 4JVN, and 4JVL) [11,12,93]. ^2^ Each SNP ID was based on NCBI dbSNP. 4-OH TCB, 4.4′-OH-3,5,3′,5′-tetrachlorinated biphenyl; TBBPA, tetrabromobisphenol A; 3-OH BDE47, 3-hydroxyl bromodiphenyl ether.

## Figures and Tables

**Table 1:** Human sulfotransferase (SULT) isoforms.

Gene ID ^1^	Locus ^2^	Alias ^1^	Number of Amino Acids ^3^	Number of Exons ^1^
SULT1A1	Chr 16p11.2	HAST1/HAST2, P-PST, PST, ST1A1, ST1A3, STP, STP1, TSPST1	295 (isoform a)	13
			217 (isoform b)	
SULT1A2	Chr 16p11.2	HAST4, P-PST, P-PST 2, ST1A2, STP2, TSPST2	295 (isoform 1)	8
			262 (isoform 2)	
SULT1A3	Chr 16p11.2	HAST, HAST3, M-PST, ST1A3, ST1A3/ST1A4, ST1A4, ST1A5, STM, TL-PST	295	8
SULT1A4	Chr 16p11.2	HAST3, M-PST, ST1A3, ST1A3/ST1A4, ST1A4, STM, TL-PST	295	8
SULT1B1	Chr 4q13.3	ST1B1, ST1B2, SULT1B2	296	10
SULT1C2	Chr 2q12.3	ST1C1, ST1C2, SULT1C1, humSULTC2	296 (isoform a)	9
			307 (isoform b)	
SULT1C3	Chr 2q12.3	ST1C3	304 (isoform 1)	10
			304 (isoform 2)	
SULT1C4	Chr 2q12.3	SULT1C, SULT1C2	302 (isoform 1)	7
			227 (isoform 2)	
SULT1E1	Chr 4q13.3	EST, EST-1, ST1E1, STE	294	9
SULT2A1	Chr 19q13.33	DHEA-ST, DHEA-ST8, DHEAS, HST, ST2, ST2A1, ST2A3, STD, SULT2A3, hSTa	285	6
SULT2B1	Chr 19q13.33	ARCI14, HSST2	350 (isoform a)	7
			365 (isoform b)	
SULT4A1	Chr 22q13.31	BR-STL-1, BRSTL1, DJ388M5.3, NST, SULTX3, hBR-STL-1	284	11
SULT6B1	Chr 2p22.2	ST6B1	304 (isoform 1)	9
			265 (isoform 2)	
SUPl1C2P1	Chr 2q12.3	SULT1C1P	pseudogene	4
SULT1C2P2	Chr 2q12.3		pseudogene	
SULT1D1P	Chr 4q13.3	SULT1D1	pseudogene	
SULT6B2P	Chr 12p12.1		pseudogene	

^1^ Information is described according to NCBI Gene. ^2^ All reference loci were based on the GRCh38 assembly. ^3^ The way to divide genes into isoform a/b or 1/2 was described in accordance with the NCBI Protein database.

**Table 2:** SULT1E1 expression in other mammalian species.

Species	RefSeq ^1^	RefSeq mRNA ^2^	RefSeq Protein ^3^	Number of Exons ^1^
*Homo sapiens* (human)	NC_000004.12	NM_005420.3	NP_005411.1	9
*Mus musculus* (mouse)	NC_000071.7	NM_023135.2	NP_075624.2	8
*Rattus norvegicus* (rat)	NC_005113.4	NM_012883.2	NP_037015.2	10
*Bos taurus* (cow)	NC_037333.1	NM_177488.3	NP_803454.2	9
*Oryctolagus cuniculus* (rabbit)	NC_013683.1	XM_002717123.2	XP_002717169.1	8
*Sus scrofa* (pig)	NC_010450.4	NM_213992.1	NP_999157.1	9
*Equus caballus* (horse)	NC_009146.3	NM_001081918.1	NP_001075387.1	8

^1^ Information is described according to the NCBI Gene database. ^2^ All reference mRNA sequences were based on the NCBI Nucleotide database. ^3^ All reference protein sequences were based on the NCBI Protein database.

**Table 3:** The nuclear receptors associated with *Sult1e1* regulation.

Gene ID	Nuclear Receptor	Species	Tissue	Reference
NR3A1	ERα	Mouse	Liver tissue	[32]
NR3C1	GR	Mouse	Liver tissue	[30]
NR1C1	PPARα	Human	Vascular endothelial cell	[24]
			Smooth muscle cell	
NR1C3	PPARγ	Human	Endothelial cell	[25]
NR1H2, H3	LXR	Mouse	Uterine	[29]
NR1H4	FXR	Human	Liver cell line	[26]
		Human	Liver tissue	[27]
NR1I2	PXR	Human	Liver cell line	[33]
		Mouse	Liver tissue	
NR1I3	CAR	Mouse	Liver tissue	[31,32]
NR2A1	HNF4α	Human	Liver tissue	[27]
NR1F1	RORα	Human	Liver cell line	[28]
		Mouse	Liver tissue	[34]

**Table 4:** Substrates of SULT1E1.

Substrate	Compound Characteristics	*K_m_*	Reference
E_1_	Agonist of ER	~0.17 µM	[39]
E_2_	Most active agonist of ER	5 ± 0.8 nM	[16]
		29 nM	[38]
EE_2_	Agonist of GPER and ER	6.7 ± 0.1 nM	[40]
DHEA	Partial agonist of AR and ER	~0.85 µM	[37]
		4.57 ± 0.07 µM	[40]
T_4_	Thyroid prohormone	22.6 ± 1.0 µM	[41]
T_3_	Receptor active iodothyronine	25.7 ± 10.4 µM	
rT_3_	Receptor inactive iodothyronine	2.15 ± 1.45 µM	
T_2_	Breakdown metabolite of triiodothyronine	4.75 ± 1.25 µM	
Apigenin	Common dietary flavonoid	5.3 ± 0.65 µM	[42]
Epicatechin	Antioxidative flavonoid	0.96 ± 0.17 mM	
Resveratrol	Antioxidative flavonoid	6.88 ± 1.12 µM	
Chrysin	Flavonoid in bee pollen or propolis	4.5 ± 0.65 µM	
Quercetin	Flavonoid in plants or fruits	2.0 ± 0.34 µM	
Fulvestrant	Steroidal ER antagonist	0.2 ± 0.02 µM	[43]
4-OH-TOR	Hydroxy metabolite of TOR (nonsteroidal agonist-antagonist of ER)	6.4 ± 0.09 µM	[44]
Troglitazone	PPAR agonist	8.5 ± 0.44 µM	[45]
Endoxifen	Active metabolite of Tamoxifen (nonsteroidal antagonist of ER)	24 ± 5 µM	[46]
4-OH TAM	Hydroxy metabolite of Tamoxifen	24 ± 5 µM	
*N*-des TAM	*N*-demethyl metabolite of Tamoxifen	96 ± 52 µM	
Tibolone	Selective tissue estrogenic activity regulator	19.5 ± 2.8 µM	[47]
3α-OH-TIB	Hydroxy metabolite of TIB	6.6 ± 2.2 µM	
3β-OH-TIB	Hydroxy metabolite of TIB	2.1 ± 0.5 µM	

*K_m_*, the constant value of Michaelis-Menten equation which is numerically equal to the substrate concentration at the half reaction rate of enzyme *V_max_*; E_1_, estrone; E_2_, estradiol; EE_2_, ethinylestradiol; DHEA, dehydroepiandrosterone; T_4_, tyroxine; T_3_, 3.3′,5-triiodothyronine; rT_3_, 3.3′,5′-triiodothyronine; T_2_, 3.3′-diiodothyronine; TOR, toremifene; ERs, estrogen receptors; GPER, G protein-coupled receptor; AR, androgen receptor; PPARs, peroxisome proliferator-activated receptors; 4-OH TAM, 4-hydroxy tamoxifen; *N*-des TAM, *N*-desmethyltamoxifen; TIB, tibolone.

## Data Availability

Not applicable.
